# Expression of Mir-21 and Mir-143 in Cervical Specimens Ranging from Histologically Normal through to Invasive Cervical Cancer

**DOI:** 10.1371/journal.pone.0028423

**Published:** 2011-12-14

**Authors:** Georgios Deftereos, Simon R. Corrie, Qinghua Feng, Janice Morihara, Joshua Stern, Stephen E. Hawes, Nancy B. Kiviat

**Affiliations:** HPV Research Laboratory, UW Medicine, University of Washington, Seattle, Washington, United States of America; University of Barcelona, Spain

## Abstract

**Background:**

MicroRNA expression is severely disrupted in carcinogenesis, however limited evidence is available validating results from cell-line models in human clinical cancer specimens. MicroRNA-21 (mir-21) and microRNA-143 (mir-143) have previously been identified as significantly deregulated in a range of cancers including cervical cancer. Our goal was to investigate the expression patterns of several well-studied microRNA species in cervical samples and compare the results to cell line samples.

**Methodology/Principal Findings:**

We measured the expression of mir-21 and mir-143 in 142 formalin-fixed, paraffin embedded (FFPE) cervical biopsy tissue blocks, collected from Dantec Oncology Clinic, Dakar, Senegal. MicroRNA expression analysis was performed using Taqman-based real-time PCR assays. Protein immunohistochemical staining was also performed to investigate target protein expression on 72 samples. We found that mir-21 expression increased with worsening clinical diagnosis but that mir-143 was not correlated with histology. These observations were in stark contrast to previous reports involving cervical cancer cell lines in which mir-143 was consistently down-regulated but mir-21 largely unaffected. We also identified, for the first time, that cytoplasmic expression of Programmed Cell Death Protein 4 PDCD4; a known target of mir-21) was significantly lower in women with invasive cervical carcinoma (ICC) in comparison to those with cervical intraepithelial neoplasia (2–3) or carcinoma *in situ* (CIN2-3/CIS), although there was no significant correlation between mir-21 and PDCD4 expression, despite previous studies identifying PDCD4 transcript as a known mir-21 target.

**Conclusions:**

Whilst microRNA biomarkers have a number of promising features, more studies on expression levels in histologically defined clinical specimens are required to investigate clinical relevance of discovery-based studies. Mir-21 may be of some utility in predictive screening, given that we observed a significant correlation between mir-21 expression level and worsening histological diagnosis of cervical cancer.

## Introduction

Early cancer detection strategies are based on the identification and validation of biomarkers which are highly indicative of disease progression from normal or precancerous tissue to early invasive cancers. MicroRNAs are a group of recently discovered short RNA species (∼21 nt) that are involved in the regulation of gene expression in a tissue-specific manner, affecting numerous cellular pathways including proliferation, differentiation and apoptosis [1]. It has been shown that microRNAs are aberrantly regulated in invasive cancer, and can act as tumor suppressors or enhancers in different tissues and environments [2]. Their recent discovery has led to a number of studies aimed at discovering novel cancer biomarkers (reviewed in [3]–[5]), however few have been validated in clinical specimens, especially those representative of pre-cancerous disease.

MicroRNAs are of increasing interest in cancer diagnostics due to the observation that a surprisingly small family of molecules can provide exquisite specificity in classifying tissue types, reflecting the developmental lineage and differentiation state. Lu *et al.* exploited this by using a microRNA panel of 217 species to classify poorly differentiated tumors with high concordance whilst a comparable mRNA panel containing ∼16,000 species failed [6]. Further studies have shown the potential for microRNAs to distinguish between cancer subtypes where histological diagnosis is complex or impossible, to diagnose tumors of unknown origin and in diagnosing cancer predisposition [4].

Investigations into the use of microRNAs as biomarkers for early cancer detection have identified surprising blood and tissue stability in contrast to mRNA [7], [8], and the development of highly sensitive and specific qPCR procedures is encouraging. However, in all cases to date, samples were extracted from patients who had already developed cancer [4], so the utility of microRNA as a marker of cancer progression from precancerous through to early invasive cancerous lesions remains unknown. Investigation of microRNA expression in samples spanning the entire range of histologically defined sample types, from normal to invasive cancer, is clearly required to properly determine the utility of microRNA expression levels as cancer biomarkers.

Cervical cancer is a disease for which stratification of histological types from normal through to invasive carcinoma is well characterized and supported by molecular techniques based on HPV genotyping. However, given the enormous success of cervical screening programs, only 35–65% CIN-3/CIS, 12–20% CIN-2 and <5% CIN-1 cases are expected to progress to more severe forms of dysplasia or invasive cancer – suggesting that markers with a higher predictive value for progression would be highly desirable [9], [10]. We therefore identified cervical cancer as an ideal test case for which to follow changes in specific microRNA expressions levels from normal through precancerous and cancerous tissues. Past studies have identified a range of aberrantly regulated microRNAs in cervical cancer cell lines, with mirs-127, 9, 203, 199a, 218, 21, 143, 205, 214,126, 15b, 16, 146a and 155 among the most common [3], [11]–[17]. However, only one of these studies included precancerous cervical specimens (CIN1-3), where high biological variability was noted in the microRNA expression levels, especially in normal samples (albeit with low sample sizes) [16].

In this study we investigated the expression profiles of two microRNAs (mir-21 and mir-143) and their previously validated target proteins in clinical samples from women with HPV infection without lesions, with histologically diagnosed pre-cancerous lesions, or with invasive cancer (ICC), as well as normal controls without HPV infection. The sequences were chosen based on previous findings from other groups identifying significant deregulation across a number of cervical cancer cell lines and some limited fresh frozen samples [12]. Our primary aim was to determine if there were significant correlations between microRNA expression and histological type which could point to a potential role for microRNA as an early marker of the oncogenic potential of pre-lesional HPV infection and/or of progression of precancer to cancer. Secondly, we compared microRNA expression in clinical samples and cancer cell lines to determine if cell lines should continue to be used as a basis for clinical investigations. We identified a significant association between mir-21 expression and histological diagnosis, but no association with mir-143. We also investigated the expression of known mir-21 target proteins (PDCD4, PTEN (phosphatase and tensin homolog), TPM1 (tropomyosin 1)) by immunohistochemistry analysis and identified a significant association between cytoplasmic PDCD4 expression and histological diagnosis. We identified discrepancies between the clinical samples and cervical cancer cell lines, suggesting that clinical samples must be investigated for validation purposes following discovery of candidate biomarkers in cell line discovery-focused studies.

## Results

### MicroRNA expression in cervical cancer cell lines

**Figure 1:**
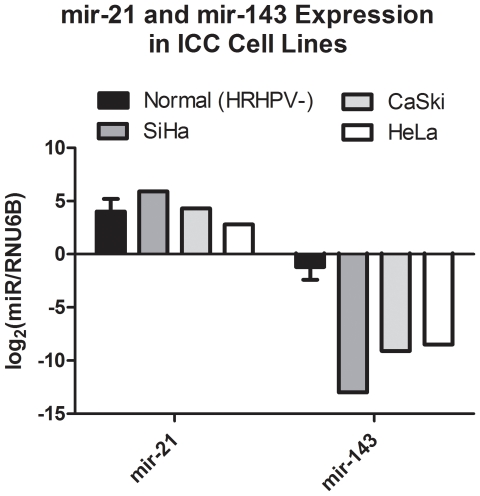
Expression profiling data for mir-21 and mir-143 in cervical cancer cell lines and normal specimens. Mir-143 was significantly down-regulated in all three cervical cancer cell lines, in agreement with previous studies. However, mir-21 was not significantly different to the normal specimens, even though in previous studies mir-21 has been observed to be significantly up-regulated in the same cell lines.

We investigated the expression of mir-21 and mir-143 in cervical cancer cell lines with respect to (Normal (HRHPV-) in order to confirm previous observations. Figure 1 shows that for all three cancer cell lines tested (SiHa, CaSki, HeLa), there was no consistent difference between mir-21 expression and that of normal samples. However, mir-143 expression of all three cancer cell lines was substantially decreased with respect to clinical samples However, we have not attached statistical analysis to these comparisons because of the different error distributions between clinical samples and cell lines, and because we would only have *n* = 3 in the case of the cell lines for comparison.

### MicroRNA expression in clinical samples

**Figure 2:**
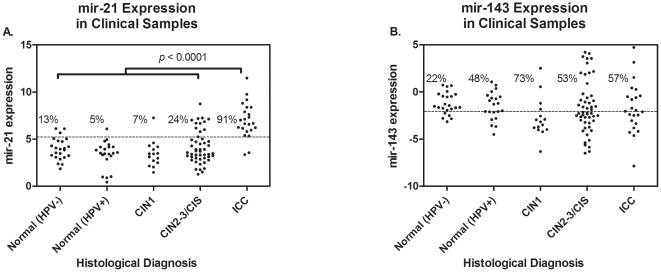
Expression profiling for (a) mir-21 and (b) mir-143 in 133 clinical samples stratified by histological diagnosis. In contrast to the cell-line data presented in Figure 1, we observed a significant association between increased mir-21 expression an worsening histological diagnosis, however no significant associations for mir-143. There was a significant difference between all histological groups (ANOVA, *p*-value<0.0001) and furthermore, women with CIS/CIN2-3 had marginally higher mir-21 expression levels compared to those classified as Normal/HRHPV+ (*p_u_* = 0.0375 *p_a_* = 0.0563).

In contrast to cell line data, we observed a significant association between mir-21 expression and histologic type in clinical samples, but no association for mir-143 (Figure 2 and Table 1). Women with ICC showed significantly increased mir-21 expression levels compared to women with CIS/CIN2-3, CIN1, normal (HRHPV+) and normal (HRHPV-) (ANOVA, *p*<0.0001). Furthermore, women with CIS/CIN2-3 had marginally increased mir-21 expression levels compared to women with CIN1 (*p_u_* = 0.14 and *p_a_* = 0.24) and normal (HRHPV+) (*p_u_* = 0.07 and *p_a_* = 0.14). We found no association between age or HRHPV status and histological type (data not shown). A cutoff of 5.06 best distinguished ICC/Normal (HPV-) populations (determined from ROC curve) and was 91% sensitive and 87% specific. Figure 2 shows how that cutoff faired for the other two histology groups. Only 5% of the samples from women who are Normal (HPV+), 7% of CIN 1, and 24% of CIN 2-3/CIS samples had MIR-21 values greater than 5.06. Interestingly, 13% of samples from women with Normal (HPV-) histology showed mir-21 expression level greater than 5.06.

**Table 1:** MicroRNA expression in FFPE cervical specimens.

	Normal (HPV-) (n = 23)	Normal (HPV+) (n = 21)	CIN1 (n = 15)	CIN2-3/CIS (n = 51)	ICC (n = 23)	*p*-Value*
**Age**	42.6±5.6	47.2±8.7	40.4±5.5	43.7±8.7	45.4±9.5	0.11
**Mir-21**	4.0±1.2	3.4±1.4	3.4±1.4	4.1±1.7	7.0±1.9	<0.0001
**Mir-143**	−1.2±1.2	−1.5±1.5	−2.7±2.1	−1.6±2.8	−1.7±2.7	0.38

*ANOVA.

### PDCD4, PTEN and TPM1 expression

**Figure 3:**
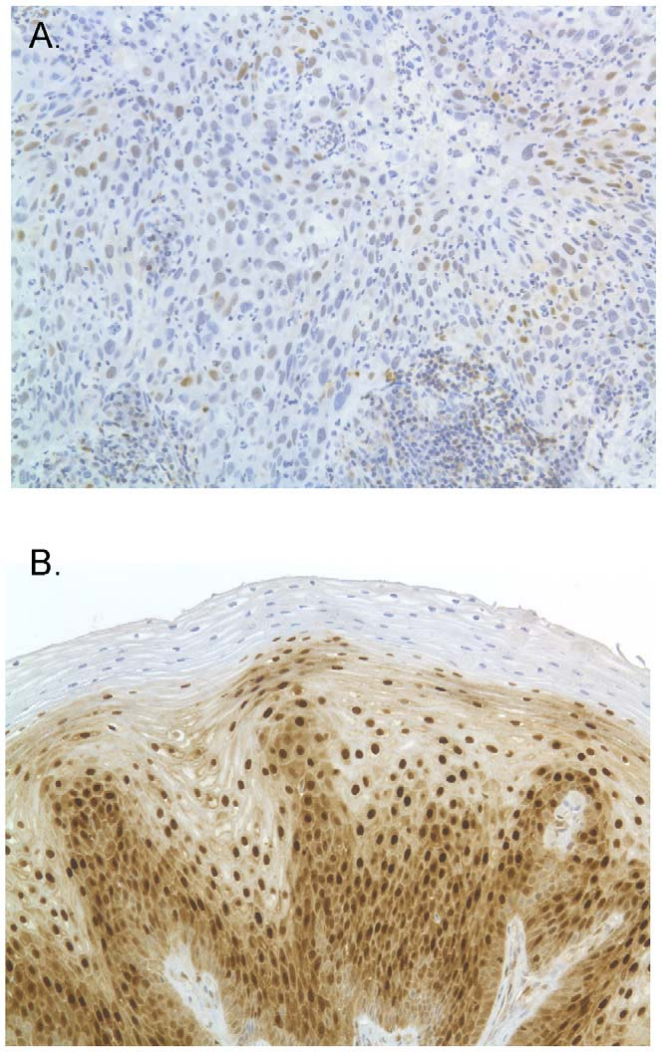
Micrographs showing PDCD4 staining of (a) Normal (HRHVP-) specimen and (b) ICC specimen.

**Figure 4:**
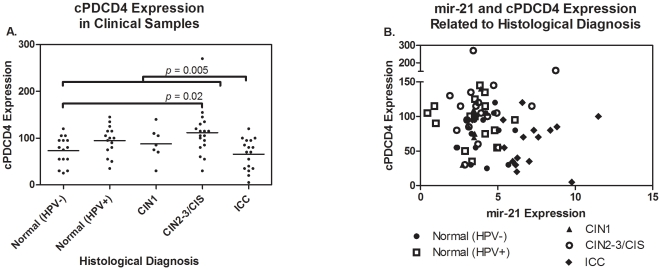
Protein expression data measured by immunohistochemistry for cytoplasmic PDCD4 stratified by histological diagnosis. Here we observed a significant association between cytoplasmic PDCD4 expression and sample histology.

In order to investigate the effect of mir-21 deregulation on tissue-specific target protein expression in clinical samples, we measured protein expression levels of PDCD4 and PTEN by immunohistochemistry (IHC) in 72 of the samples tested for mir-21 expression. TPM1 expression was evaluated on a subset of samples, however the level of TPM1 expression in cervical epithelium was found to be below the threshold of detection by immunohistochemistry. We observed a significant association between cytoplasmic PDCD4 (cPDCD4) expression and sample histology (Table 2, Figure 3 and Figure 4). Samples from women with ICC had significantly lower cPDCD4 expression compared to those with CIN2-3/CIS (pu = 0.0005 or pa = 0.003) and marginally lower expression compared to (HRHPV+) (*p_u_* = 0.03 or *p_a_* = 0.11). Women in the normal (HRHPV-) group had significantly lower cPDCD4 expression compared to CIS/CIN2-3 (*p_u_* = 0.005 or *p_a_* = 0.02). There also appeared to be a marginal decrease in nuclear PDCD4 (nPDCD4) in women with ICC in comparison to those with CIN2-3/CIS. There was no significant correlation between PDCD4 expression (nuclear or cytoplasmic) and mir-21 expression in these samples, however we observed a general grouping of ICC with high mir-21 but not PDCD4, and CIN-2,3/CIS with higher PDCD4 but non-elevated mir-21 (Figure 4). There was also no association between PTEN protein expression and sample histology or microRNA expression. We considered analyzing IHC by both the frequency of the maximum intensity score and the frequency of the predominant intensity score. However, we observed similar results compared to the composite score analysis and determined there is no advantage for using the frequency of intensity scores over the composite scores.

**Table 2:** Protein expression results for 72 clinical samples.

	Normal (HPV-) (n = 15)	Normal (HPV+) (n = 15)	CIN1 (n = 7)	CIN2-3/CIS (n = 18)	ICC (n = 17)	p-value*
**Age**	42.1±4.9	46.6±9.3	42.3±4.8	45.5±10.6	46.4±10.7	0.55
**nPDCD4**	122.0±47.9	143.0±49.3	124.3±51.8	152.8±61.4	110.9±40.7	0.13
**cPDCD4**	73.3±30.2	94.7±31.4	87.9±35.0	111.9±50.8	65.6±33.1	0.006
**nPTEN**	70.3±40.8	81.7±35.6	44.3±37.2	68.1±28.5	64.4±46.9	0.32
**cPTEN**	64.0±31.8	69.0±29.3	47.9±43.1	68.3±28.2	69.7±36.0	0.62

*ANOVA.

## Discussion

While microRNA expression profiling has been shown to be highly specific for cancer typing, tumor diagnosis and identifying tumors of unknown origin, it is not yet clear if microRNAs can perform as markers of disease progression – a crucial requirement for identifying those conditions which require treatment. Ideally, such markers of progression would show systematic changes in expression in precancerous tissues, which correlate with disease progression to invasive cancer. Clearly, this requires access to large numbers of clinical samples which cover the range of histologically defined tissue types involved in disease progression. In this study we investigated the expression levels of two key MicroRNAs identified in the literature (and their validated protein targets) in cervical tissue stratified by histological diagnosis in order to determine their utility as biomarkers for early cancer diagnosis. We found that mir-21 expression was significantly increased only in ICC with respect to other clinical samples and that mir-143 expression did not correlate with histology at all. Cervical cancer cell line results, while consistent with previous studies on the same cell lines, appeared to give discordant microRNA expression results in comparison to clinical samples. Interestingly, PDCD4 protein expression increased in precancerous lesions then decreased significantly in ICC, possibly as an effect of mir-21-induced silencing. These results highlight the need for further studies focusing on tissue profiling in preference to cell lines, where expression levels of candidate MicroRNA biomarkers can be assessed in pre-cancerous disease, where their utility in cancer diagnostics would prove most beneficial. We identified key inconsistencies in microRNA expression profiles in our clinical specimens as compared to cervical cancer cell lines. However, recent investigations show significant differences in microRNA expression based on cell culture conditions suggesting that cell line experiments may not be particularly informative when searching for clinically relevant microRNAs [12]. Cheng *et al.* 2005 [18] showed significant increase in HeLa cell growth and proliferation in response to inhibition of mir-21, whereas Yao *et al* 2009 [14] showed significant decrease in cell growth and proliferation for the same treatment. Wang *et al* 2008 [12] further showed that changing culture conditions from a monoculture to a suspension raft culture led to a completely different set of significantly deregulated microRNAs. Similar discordant results have been seen in other studies investigating the relevance of cell culture models in comparison to *in vivo* tissue of spheroidal cultures [19], [20]. Also, other gene/protein expression differences have recently been identified, including the presence of WNT5A expression in gastric cancers (not expressed in cell lines) [21] and differences in DNA methylation [22] and gene expression [23] signatures between colorectal carcinomas and colon cancer cell lines. Clearly, a key problem with the cell line approach is that potential biomarkers are identified in cells immortalized from invasive cancers – it is often completely unknown if these biomarkers can be detected in pre-cancerous disease, which requires analysis of clinical samples. A further complication in the current study may be the racial background (West African) in comparison to cancer cell lines which are Caucasian – however little information is known about such variation.

Increased mir-21 expression has been identified in a large number of tumor types [24]–[29] and in this study has shown significant association with the development of histologically-defined ICC. Furthermore, our results show that mir-21 may be of some utility in predicting the progression of pre-cancerous lesions into ICC, as women with high mir-21 values may be expected to progress. However, further studies involving larger sample sizes and patient follow-up are required to investigate this aspect, as there are clearly insufficient data for proper testing of this hypothesis by splitting into training and testing sets. Furthermore, while the mir-21 expression levels of women with normal pathology with and without HRHPV infection are not statistically different, the HRHPV- group showed approximately double the number of samples above the optimal cut-off compared to the HRHPV+ group. The significance of this result is again difficult to quantify due to the relatively low sample sizes involved, but it may indicate that a panel of biomarkers may be necessary to help reduce false positives.

A number of studies have identified mir-143 as significantly underexpressed in cancer, including breast and cervical cancers [12], [17], [30]. Whilst we also observed significant underexpression in cervical cancer cell lines with respect to normal (HRHPV-) samples, we did not find any statistically significant associations with respect to clinical samples from women across a wide spectrum of neoplastic disease. Previous studies investigating microRNA expression in cervical samples again show conflicting results. Lee *et al* 2008 [11] did not include either mir-21 or mir-143 in their initial screening experiments and as such did not identify these species as being involved in cancer development; this highlights a clear challenge in identifying potential biomarkers, in cases where leading candidates are ignored. However, Wang *et al* 2008 [12] suggested that reduction of mir-143 was required for tumor development whilst Lui *et al* 2007 [17] showed mixed results for mir-143, mainly due to the low level of expression detected in either normal or cancerous samples. Inconsistencies may be due to the relatively low level mir-143 expression in cervical tissues, which may require a more precise extraction method (*e.g.* laser microdissection) for tumor extraction – none of the studies listed here used such methods.

Previous studies revealed that PDCD4, PTEN and TPM1 are all transcripts targeted by mir-21 in various tissues [14], [17], [24], [26], [31], [32] and that PTEN is progressively underexpressed from normal tissue through to cervical cancer [33]–[35]. One recent study has also reported reduced PDCD4 expression in cervical cancer cell lines [14] which is in agreement with our findings in the clinical samples examined in this study. In fact recent studies have demonstrated not only a direct relation between PDCD4 expression and tumor progression (Wei *et al*, 2009 [36]) but also the key role of PDCD4 and mir-21 in apoptotic inflammatory response (Sheedy *et al*, 2009 [37]). Moreover, recent cell model studies support suggest a role for loss of PDCD4 expression in increased sensitivity of cells to agents that cause DNA damage (Singh *et al*, 2009 [38]), all of which would be expected to provide favourable conditions for tumor growth. Mice expressing high levels of PDCD4 are also more resistant to tumor growth in comparison to normal controls. We hypothesise that cPDCD4 expression (and, to a lesser extent, nPDCD4 expression) is increased in pre-cancerous specimens due to increased apoptosis, followed by a sharp decrease in expression for ICC specimens, where apoptotic mechanisms are subverted. Similar trends have been observed for other tumor suppressor genes in relation to oncogenesis and apoptosis [39], [40]. Interestingly, we did not identify any correlation between PTEN expression and histological diagnosis; loss of expression has been noted previously, and we have observed lower expression in a breast cancer study using the same methods (data not shown).

In this study we have found that the expression level of mir-21 is significantly correlated with histological diagnosis of ICC. A known target of mir-21, PDCD4 was also downregulated but was not significantly correlated with mir-21 expression. Expression of mir-143 was not correlated with histological diagnosis of clinical samples, however it was significantly down-regulated in cervical cancer cell lines. These findings are important in the quest to gauge the utility of MicroRNA biomarkers for diagnostic applications, a key component of which is to measure expression levels in precancerous tissues as well as normal and invasive cancers. Clearly, future work must focus on clinically relevant samples, in which predictive markers expressed in pre-cancerous tissue can be identified for further validation.

## Materials and Methods

### Selection of microRNAs

MiRs investigated in this study were chosen based on previous observations in the literature (Table 3). We specified that each microRNA should also have an experimentally verified mRNA target or set of target genes and at least one of the predicted targets had to be reported as involved in oncogenesis and progression in cervical or other neoplasms (based on sequences in the miRBase database [41]–[43]). MiR-21 is reported to be overexpressed in a number of cancers [29], [44] and targets transcripts of the *PTEN* [24], *TPM1* [26] and *PDCD4* [14], [31], [32] genes, whilst miR-143 is underexpressed in many cancers and targets *ERK5* transcripts [17], [29], [30]. Both mir-21 overexpression and mir-143 underexpression have been observed in cervical cancer cell lines and clinical specimens [12], [17].

**Table 3:** Cancer associations for MicroRNAs used in this study.

MicroRNA	Cancer Association	Validated Target	Reference
**Mir-21**	Cervical	PDCD4	[14]
	Colon	PDCD4	[31]
	Prostate	MARCKS	[50]
	Stomach	PTEN	[51]
	Breast	PDCD4, TPM1	[26], [32]
	Liver	PTEN	[24]
	Lung, pancreas	–	[17], [29], [44]
**Mir-143**	Breast	ERK5	[30]
	B-cell tumors	ERK5	[52]
	Cervical, prostate, colon	–	[17], [29], [53], [54]

### Samples

Clinical specimens were collected from the Dantec Oncology Clinic, Dakar, Senegal from studies of cervical cancer [45]; all study procedures were approved by the institutional review boards of the University of Washington (Seattle, WA) and University of Dakar (Dakar, Senegal) and informed written consent was provided by study participants. Histological diagnoses were produced at the University of Washington, Department of Pathology, Harborview Medical Center, Seattle, WA by a pathologist (NBK). Samples collected included 142 cervical biopsies preserved as FFPE tissue blocks. HPV genotyping was performed as described previously [46], with High-Risk HPV (HRHPV) subtypes HPV16/18/26/31/33/35/39/45/51/52/53/56/58/59/66/67/68/73/82 as defined by Munoz *et. al.* 2006 [47]. Clinical specimens were grouped according to histological diagnosis, with groups defined by treatment regimens per current guidelines [48]. Normal (HRHPV-) identified those women with a normal histology and a negative test result for HRHPV (*n* = 23). Normal (HRHPV+) indicated those women with a normal histology and a positive test for HRHPV (*n* = 21). Mildly dysplastic CIN1 lesions (*n* = 15) typically spontaneously regress and women with these lesions are usually followed with repeat Pap smear. Women with precancerous CIN2-3/CIS lesions (*n* = 51) would all be referred for treatment to prevent development of invasive disease ICC (*n* = 23). These groupings allowed us to determine if the microRNA biomarkers under investigation could identify various stages of neoplastic disease which require different clinical management. The average age of the 133 subjects from who clinical specimens were used was 44 ranging from 29 to 72 years. All four histologic groups were frequency matched on age in order to achieve similar age distributions and means, which ranged between 43 and 45 years. Cell lines and culture media were purchased from ATCC and Gibco (Invitrogen, Carlsbad, CA).

### MicroRNA qPCR expression analysis

Extraction of microRNA from blocks was performed using the RecoverAll Total Nucleic Acid Isolation Kit (Applied BioSystems, Foster City, CA), according to manufacturer's instructions. The only alteration was that for cell line extraction, the deparaffination steps were omitted on the advice of Applied Biosystems staff. Reverse transcription (RT) reactions and quantitative polymerase chain reactions (qPCR) were performed using the MicroRNA TaqMan Reverse Transcription Kit and the TaqMan MicroRNA Assays (Applied BioSystems) for mir-21, mir-143 and RNU6B control shRNA. RT and qPCR for the RNU6B control shRNA analysis was performed side-by-side with the mir-21 and mir-143 reactions for each sample. A standard curve was generated by extracting total RNA from VK2 cells and performing RT reactions on 10-fold dilutions, subsequently including each serial dilution in each qPCR run, throughout the whole study.

### Immunohistochemistry

PDCD4 rabbit polyclonal antibody and TPM1 mouse monoclonal antibody was purchased from Abcam (Abcam Inc., Cambridge MA, ab51495, ab17784 respectively), PTEN mouse monoclonal antibody was purchased from Dako (Dako Corporation; Carpinteria, CA, clone 6H2.1) Four-micron sections of formalin-fixed paraffin-embedded tissue were cut and placed on Superfrost Plus microscope slides. The tissue sections were deparaffinized and rehydrated through a series of graded alcohols. Antigen retrieval was carried out with Tris/EDTA pH 9.0 buffer in a tabletop autoclave. Endogenous peroxidase activity was blocked with 3% H_2_O_2_. Primary antibodies were varied to determine the optimal antibody concentration resulting in the most intense staining with no background. Antibodies for PDCD4 were diluted 1∶2000 and PTEN antibody was diluted to 1∶500 and applied to each slide. TPM1 antibody was diluted to 1∶100.The slides were washed in ImmPRESS (Vector Laboratories; Burlingame, CA), then an anti-mouse (for PTEN and TPM1) and an anti-rabbit (for PDCD4) IgG polymerized reporter enzyme was applied to each tissue. Color development was accomplished by incubation in diaminobenzidine (Dako). The slides were counterstained in hematoxylin (Dako), dehydrated through graded alcohols, cleared in xylene, and coverslipped with permanent mounting media. A negative tissue control was run on each tissue, where antibody diluent buffer was substituted for the primary antibody. In addition, positive control tissues known to express the antigen in question were run in conjunction with each batch of slides. PDCD4 and PTEN staining was evaluated semi-quantitatively using the H-score scoring method [49]. This method of quantification takes into account the staining intensity and the percentage of cells staining at a particular intensity. Staining intensity was scored as 0 (no staining), 1 (weak staining), 2 (moderate staining), 3 (intense staining). Each intensity score was multiplied by the percentage of cells staining at that level. The resulting values were summed to yield a composite score ranging from 0 to 300. PDCD4 and PTEN expression was evaluated in both the nuclear and cytoplasmic compartments.

### Statistical Methods

Taqman qPCR reactions were performed in triplicate redundancy and the Ct values converted to a ratio with respect to the RNU6B control. Non-cancer controls were frequency age-matched to cases with invasive cancer. Values of both microRNAs were log_10_-transformed in order to normalize the distribution of these measurements for all statistical analyses. ANOVA was used to compare all histologic groups with respect to the continuous factors of interest. P-values are reported as unadjusted (*p_u_*) and adjusted for multiple comparisons using false discovery rates (*p_a_*). Receiver Operating characteristic (ROC) curves were used to assess the predictability of cervical disease progression. Optimal cutoffs were determined to best distinguish Normal (HPV-) women from women with ICC. A two-sided 0.05 test level determined statistical significance for all analyses. All analyses were conducted using SAS version 9.2 (SAS Institute Inc., Cary, NC).
